# Learning Deficits and Attenuated Adaptive Stress Response After Early-Life Seizures in Zebrafish

**DOI:** 10.3389/fnins.2022.869671

**Published:** 2022-04-22

**Authors:** Harsimran Singh, Alfonsina Ramon, Dana Finore, Kaleigh Burnham, Scott McRobert, Jocelyn Lippman-Bell

**Affiliations:** ^1^Department of Bio-Medical Sciences, Philadelphia College of Osteopathic Medicine, Philadelphia, PA, United States; ^2^Department of Biology, Saint Joseph’s University, Philadelphia, PA, United States

**Keywords:** early-life seizures, PTZ animal model, larval zebrafish, seizure comorbidities, adaptive stress response, zebrafish behavioral model

## Abstract

Early-life seizures (ELS) are often associated with the development of cognitive deficits. However, methods to predict and prevent these deficits are lacking. To increase the range of research models available to study cognitive consequences of ELS, we investigated whether seizures in larval zebrafish (*Danio rerio*) lead to behavioral deficits later in life. We thus modified the existing pentylenetetrazole (PTZ)-induced seizure model in larval zebrafish, exposing zebrafish to PTZ daily from 5 to 7 days post-fertilization (dpf). We then compared later-life learning, social behavior (shoaling), and behavioral and chemical measures of anxiety in the PTZ-exposed zebrafish (PTZ group) to that of naïve clutchmates (untouched controls, UC) and to a second control group (handling control, HC) that experienced the same handling as the PTZ group, but without PTZ exposure. We observed that only the PTZ group displayed a significant deficit in a y-maze learning task, while only the HC group displayed a social deficit of decreased shoaling. HC fish also showed an increased frequency of behavioral freezing and elevated cortisol responses to netting, heightened stress responses not seen in the PTZ fish. Since mild stressors, such as the handling the HC fish experienced, can lead to learned, advantageous responses to stress later in life, we tested escape response in the HC fish using an acoustic startle stimulus. The HC group showed an enhanced startle response, swimming significantly farther than either the PTZ or UC group immediately after being startled. Taken together, these results indicate that seizures in larval zebrafish impair learning and the development of an adaptive, heightened stress response after early-life stress. These findings expand the behavioral characterization of the larval zebrafish seizure model, strengthening the power of this model for ELS research.

## Introduction

Epilepsy is the most common pediatric neurological condition, with seizures often occurring as early as the first year of life (Aaberg et al., 2017). An estimated 30–50% of children with epilepsy also have comorbid psychiatric conditions, and cognitive deficits ranging from subtle changes in IQ scores to severe cognitive dysfunction (Holmes, 2016; Sillanpää et al., 2016). Research in rodent models has uncovered multiple mechanisms by which early-life seizures (ELS) might lead to cognitive deficits (Bernard and Benke, 2015). Still, we currently can neither predict nor prevent these deficits.

The larval zebrafish pentylenetetrazol (PTZ)-induced seizure model has added a great deal of insight into ELS seizure mechanisms since it was first described in 2005 (Baraban et al., 2005). Zebrafish have a high level of gene homology with humans (Howe et al., 2013), breed quickly, produce large numbers of offspring, and absorb chemicals such as chemoconvulsants easily through their gills, avoiding some of the stressful aspects of seizure induction methods used in rodent models. Specific to cognitive and behavioral investigations, zebrafish are social animals with a demonstrated capacity for learning (Cognato Gde et al., 2012), and are useful for modeling neurodevelopmental disorders (de Abreu et al., 2020). In adult zebrafish, chemically-induced seizures can result in deficits in learning six to 24 h after seizure induction, and increased anxiety and aggression 30 days after seizures (Lee et al., 2010; Canzian et al., 2019; Budaszewski Pinto et al., 2021). However, it is unclear how much of the data collected using the larval seizure model can be applied to the cognitive and behavioral consequences of ELS since long-term behavior and cognition following larval seizures have not yet been characterized.

Therefore, to increase the range of research models available to study cognitive consequences of ELS, we investigated whether seizures in larval zebrafish (*Danio rerio*) lead to cognitive and behavioral deficits later in life. After inducing seizures by PTZ exposure from 5 to 7 days post-fertilization (dpf), we assessed three behavioral and cognitive outcomes that can be impaired after seizures in humans and rodents: learning and memory (Hermann et al., 2008; Lugo et al., 2014), social behavior (Lippman-Bell et al., 2013; Dal Canto et al., 2018), and anxiety (Castelhano et al., 2013, 2015; Dal Canto et al., 2018). Specifically, we measured performance in a y-maze novel preference recall task to assess learning and memory (Cognato Gde et al., 2012), shoaling behavior to assess social behavior (Ledesma and McRobert, 2008; Miller and Gerlai, 2011), and freezing behavior, acute cortisol levels after a mild stressor, and glucocorticoid receptor gene expression as multifactorial measures of anxiety (Egan et al., 2009; Lugo et al., 2014; Tran et al., 2014). To capture developmental delays in learning and social behavior, we assessed these behaviors at the first ages at which control zebrafish consistently demonstrated the behavior measured by the task. For shoaling, this develops in juveniles near 1 month of age (Buske and Gerlai, 2011). For the y-maze, we first observed consistent recall of a novel arm in control fish older than 3 months (adults).

We found that zebrafish that experienced PTZ-induced seizures at 5–7 dpf showed learning deficits in the y-maze, without changes in social behavior or anxiety compared to naïve clutchmates (untouched controls, UC). However, the fish that were handled like the PTZ fish from 5–7 dpf but not exposed to PTZ (handling controls, HC) showed a heightened acute stress response not exhibited in either the UC or PTZ fish, as well as a faster escape response. From these results, we concluded that seizures in larval zebrafish impair learning and the development of an adaptive heightened stress response after experiencing mild early-life stress. These findings provide an important addition to the existing larval zebrafish seizure models, supporting the use of larval zebrafish for future research into the cognitive consequences of ELS.

## Materials and Methods

### Animal Husbandry

Zebrafish of the Zebrafish International Resource Center WIC (ZIRC WIC) lineage were bred and housed at Philadelphia College of Osteopathic Medicine (PCOM) laboratory animal research facilities (Philadelphia, PA, United States). Animals were housed in 3 L tanks on a 14:10 h light dark cycle. Water was recirculated and conditioned using a Pentair G-Hab (Cary, NC, United States) or Tecniplast Blue Active (Milan, Italy) aquatic habitat. Conductivity was maintained between 500 and 1,000 mΩ–1, pH between 7.0 and 7.5, and temperature between 27 and 28°C. Zebrafish were fed GEMMA Micro (Westbrook, ME, United States). Animals used for behavioral experiments were kept as breeders or euthanized humanely. All protocols were carried out in accordance with the regulations set by the PCOM and St. Joseph’s University Institutional Animal Care and Use Committees (IACUC).

### Seizure Induction

Our pilot studies using the single-day PTZ-induced seizure model first described by Baraban et al. (2005) did not show significant effects on y-maze or social behavior (Supplementary Figure 1). Therefore, we modified the previously published seizure model by increasing seizure induction sessions from one to three (once daily for 3 days), and decreasing the concentration of PTZ (MilliporeSigma, Burlington, MA, United States) to compensate for the increased amount of time the fish were exposed to PTZ. Specifically, at 5 days post-fertilization (dpf), zebrafish were randomly divided into three groups: untouched controls (UC), handling controls (HC), and seizure (PTZ group) and kept in 250 mL beakers with aquarium system water. HC and PTZ fish were pipetted individually into 24-well plates with 500 uL tank water, where they acclimated for 20 min while UC group remained in the incubator undisturbed. Temperature within the experimental arena was kept at 26–28*^o^*C using a heating pad under the arena. Next, 500 uL of clean tank water was added to the HC group and 500 uL of 10 mM PTZ mix with aquarium water was added to the PTZ group (final concentration of 5 mM PTZ). Behavior of HC and PTZ fish was recorded using EthoVisionXT software with a GigE camera (Noldus, Leesburg, VA, United States) for 40 min. Fish were returned to their home beakers after another 40 min and kept in a 28*^o^*C incubator overnight. This procedure was repeated on 6 and 7 dpf. During PTZ exposure, zebrafish showed Stage 2 seizure behavior typical of what has been reported in single day seizure models. This included circular “whirlpool” swimming (Baraban et al., 2005; Bandara et al., 2020), increased distance moved, increased overall swim velocity, and increased time spent swimming at high speed (≥2 cm/s) (Winter et al., 2017), compared to HC fish not exposed to PTZ (Supplementary Figure 2). Fish that did not show Stage 2 seizure behavior at 5 dpf (approximately 1–2%) were removed from the study. All three groups of zebrafish were transferred to the larger rack system tanks on 8 dpf by gently pouring the entire contents of the beaker (water and fish) into the tank to avoid additional handling. Groups remained separated for identification purposes.

### Y-Maze

**FIGURE 1:**
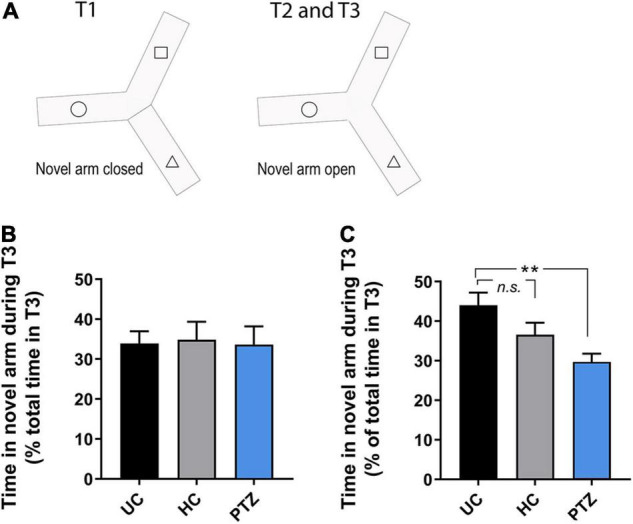
Zebrafish that experienced seizures as] larvae showed learning deficits at 12–14 weeks of age. **(A)** Diagram of the y-maze paradigm. The arm with the dotted line represents the randomly chosen starting arm; the blocked off arm represents the novel arm. Fish freely explored the first two arms during Trial 1, and all three arms in Trial 2. Fish were then removed from the maze for the designated inter-trial interval (ITI), then returned to the starting arm to explore all three arms for Trial 3. **(B)** In 9.5-week old zebrafish, no experimental group showed robust, consistent recall of the novel arm in Trial 3, even with a shorter ITI of 60 min [*p* = 0.975; *F*_(2,33)_ = 0.025 by ANOVA; *n* = 13 UC, 12 HC, 11 PTZ]. **(C)** By 12–14 weeks old, the untouched controls (UC) group showed a strong recall of the novel arm at 90 min ITI. However, the performance of zebrafish in the pentylenetetrazole (PTZ) group was significant impaired compared to the UC controls [*p* = 0.004, *F*_(2,40)_ = 6.223 by ANOVA; *post-hoc* PTZ vs. UC *p* = 0.002. ** indicates *p* < 0.01.

To assess learning and memory, we used a y-maze task, as described by Cognato Gde et al. (2012), in fish 8–14 weeks post-fertilization. The y-maze consisted of an acrylic apparatus with three arms of equal dimensions, 25 cm long, 8 cm wide, and 15 cm high. The external surface of each arm was covered with a black adhesive film to block outside stimuli. Each arm was lined with white cut-outs of squares, triangles, or circles (one shape per arm). These specific shapes were selected because zebrafish exhibit approximately the same amount of preference for each and did not show signs of aversion in previous studies (Cognato Gde et al., 2012). The y-maze apparatus was filled with 3 L of tank water, with all shapes below the waterline. An opaque black partition was used to block off one of the arms at random. One of the remaining two open arms was randomly chosen as the starting arm. The experiment consisted of three trials; acclimation (Trial 1), observation (Trial 2), and recall (Trial 3). In this task, the zebrafish acclimate to a test apparatus with two of the three arms open for exploration (Trial 1; Figure 1A). Next (Trial 2), the blockade to the third arm is removed, and the fish continued exploring for 5 min. During Trial 1, a zebrafish was placed in the starting arm and allowed to freely explore the two open arms for 20 min while acclimating to the test apparatus (Figure 1A). During trial 2, the partition was lifted, supplying novel stimuli (space and visual shape), and the fish was allowed to explore for additional 5 min (Figure 1A). At the end of trial 2, the fish was removed from the maze and transferred to a 2 L tank in a 27.5°C incubator for a 90-min inter-trial interval (ITI). During trial 3, the fish was returned to the original starting arm, with all three arms open, and given an additional 5 min to explore freely. During each trial, the fish was recorded and movement was tracked using a GigE camera and Noldus EthoVision XT software. Time spent in each arm was measured as a percent of the total time in the trial. Any fish that spent less than 15% of the total time in the novel arm in T2 were excluded from T3 final analysis (Supplementary Figure 3; total excluded = 1 UC, 3 HC, 4 PTZ).

### Shoaling (Social) Behavior

At 3 weeks of age, HC, UC, and PTZ zebrafish were transported from PCOM to St. Joseph’s University. Fish were allowed to acclimate to their environment for 1 week, then tested for social behavior at 28–32 dpf using a shoaling assay (Way et al., 2016). The shoaling assay apparatus consisted of a plastic tank (30 mm-wide × 180 mm-long × 40 mm-deep) divided into three equal compartments with clear plastic panels. Three preference zones within the central compartment were delineated by vertical lines marked the outside surface of the tank, 20 mm from each end compartment. Five target zebrafish of the same age and lineage were added to either the left or right compartment at random. The preference zone closest to the target fish was denoted as +1. The center was preference zone 0 and the preference zone furthest from the target fish was –1. For each assay, a single 30–40 dpf zebrafish was added to preference zone 0 and allowed to acclimate to the tank for 1 min. After 1 min, fish were recorded for the following 10 min using an Apple iPad. Fish then acclimated for an additional 49 min in the testing apparatus, for a total time in-tank time of 1 h, then recorded for another 10 min. Videos were transferred to Noldus EthoVision XT software for tracking the time spent in each sub-zone by the test fish. Time spent in the +1 zone was considered a positive shoaling response or normal social behavior, while time spent in zones 0 or –1 was considered a negative shoaling response or abnormal social behavior. Shoaling baseline varied by clutch, therefore, data were normalized to the average shoaling of the UC group for each clutch at 1 min.

### Stress/Anxiety Behaviors

Freezing behavior was used as a measure of anxiety (Egan et al., 2009). Freezing was assessed using Noldus Ethovision XT behavioral tracking software to quantify cumulative time immobile, and defined as a less than 0.5% change in body pixels between frames. This was then normalized to the total time the fish was tracked, resulting in percent time immobile. Measurements were taken from 28–32 dpf zebrafish during the shoaling task and from zebrafish 12–14 week post-fertilization during the y-maze task. Data were analyzed by ANOVA on GraphPad Prism software.

### Molecular Assays of Stress

#### Cortisol Assay After Netting

At 30 and 60 dpf, fish were exposed to a mild physical stressor, “netting,” for 2–3 s. Holding a zebrafish in a net significantly increases acute cortisol levels in wild-type fish, in a duration-dependent manner (Ramsay et al., 2009). Two to three seconds of netting should be a very mild stressor for control wild-type zebrafish (Tran et al., 2014). After netting, fish were immediately euthanized using pH neutral 250 mg/L tricane solution. After euthanasia, fish were homogenized, and cortisol was extracted using diethyl ether (Egan et al., 2009). Samples from pooled fish (five/sample) normalized by mass. One mL of chilled PBS was added and samples were homogenized for 30 s using a PRO Scientific PRO200 handheld homogenizer. Next, diethyl ether was added to each sample. Samples were then centrifuged at 10,000 g for 15 min. The floating organic layer was removed from each sample and stored in glass vials. An additional 5 mL of diethyl ether was added, then samples were centrifuged for another 15 min. The organic layer was removed and stored, and the aqueous layer was discarded. The samples evaporated overnight, and were then reconstituted with 1 mL of PBS. After 24 h, samples were analyzed using a Salimetrics, Carlsbad, CA, United States, salivary cortisol ELISA kit per manufacturer protocol. Plates were read using a Bio-Rad iMark microplate absorbance reader at an excitation wavelength of 450 nm. Samples were run in triplicate; absorbance values were averaged. Cortisol concentration was calculated based on a standard curve. Due to clutch-to-clutch variations in cortisol levels, values were normalized to UC’s for each individual clutch.

#### Glucocorticoid Receptor Qualitative Real-Time Polymerase Chain Reaction After Netting

Qualitative real-time polymerase chain reaction (qRT-PCR) was used to examine glucocorticoid receptor gene expression. 30 and 60 dpf were euthanized with tricaine, snap frozen in liquid nitrogen, and pooled for mRNA analysis (five/sample). mRNA isolation and qPCR were based on published protocols (Lan et al., 2009) and run on a StepOnePlus Real-Time PCR system (Applied Biosystems, Waltham, MA, United States). Two-hundred ng of RNA was used for each reaction, combined with the Luna-Universal one-step RT-PCR master mix (New England Biolabs, Ipswich, MA, United States), SYBR green as the reporter dye, and ROX as a reference dye. Expression of glucocorticoid receptor 1a (nr3c1) was assessed using the ΔΔCt method. Elongation factor 1a (Elf1a) was selected as a housekeeping control due to its stable expression during development (McCurley and Callard, 2008). Forward and reverse primers were used for all genes and reactions were run in triplicate (Supplementary Table 1).

### Acoustic Startle Response Assay

To assess escape response, we used the acoustic startle response (ASR) assay described by Best et al. (2008). Larval zebrafish (12–13 dpf) acclimated in 12-well plates within a temperature and sound controlled DanioVision, Noldus, Leesburg, VA, United States, chamber for 25 min prior to exposure to the acoustic stimulus. The stimulus consisted of an approximately 83 dB tap, a stimulus that was loud enough to cause a startle response in some but not all (91.7%) of UC fish tested. The onset of the tap was controlled using EthoVision software. For the duration of the experiment, fish were recorded using a Basler acA1300-60 gm GigE camera. Pre-tone baseline movement was quantified using the first 20 s prior to the first tap for each fish during the acclimation period. The acoustic startle response for each larval zebrafish was quantified by measuring the distance moved (cm) for 1 second from the beginning of each tapping stimulus. HC and PTZ larvae responses were compared to that of UC larvae, before and after the tap, by 2-way repeated measures ANOVA.

### Statistical Analyses

Statistical tests were performed using GraphPad Prism software, San Diego, CA, United States, version 9.2. All data were tested for normality. Data and graphs are represented as mean ± standard error unless stated otherwise. Significant differences were defined as *p* < 0.05.

## Results

### Adult Zebrafish That Experienced Larval Seizures Displayed Learning Deficits

Zebrafish tend to explore novelty and will investigate novel 2-D shapes and colors on the walls of their tank (Cognato Gde et al., 2012). The Y-maze task takes advantage of this behavior, allowing for quantification of zebrafish learning and memory (Cognato Gde et al., 2012). Most zebrafish preferred the novel arm in Trial 2 regardless of the experimental group (Supplementary Figure 1). For trial 3, the expected response in controls is recall and preference for the novel arm (Cognato Gde et al., 2012). In 8–9 weeks old fish, the control fish did not show consistent evidence of robust recall, even at a shorter 60-min inter-trial interval (ITI) (*p* = 0.975; *F*_(2,33)_ = 0.025 by ANOVA; *n* = 13 UC, 12 HC, 11 PTZ; Figure 1B). Therefore, we next tested 12–14 week old zebrafish. We found that the UC fish demonstrated the expected strong preference for the novel arm in Trial 3, spending an average of 44 ± 3.2% of the total time of Trial 3 in the novel arm. However, the PTZ fish showed a significant deficit in recall, spending less than a third of the total time of Trial 3 in the novel arm (29.7 ± 2.1%; *p* = 0.004 by ANOVA, *F*_(2,40)_ = 6.223, with *p* = 0.002 by Dunnett’s multiple comparisons *post-hoc* test of UC vs. PTZ; Figure 1C). The HC group showed a slight decrease in recall that was not significantly different from the UC recall and still spent greater than one-third of trial 3 in the novel arm (36.6 ± 3.0%; with *p* = 0.15 by Dunnett’s multiple comparisons *post hoc* test of UC vs. HC; *n* = 18 UC, 12 HC, 13 PTZ fish). The decreased y-maze recall of the PTZ group demonstrates that seizures in larval zebrafish can have long-term effects on learning.

### Handled Controls Displayed Decreased Social Behavior Not Observed in Fish That Experienced Seizures

**FIGURE 2:**
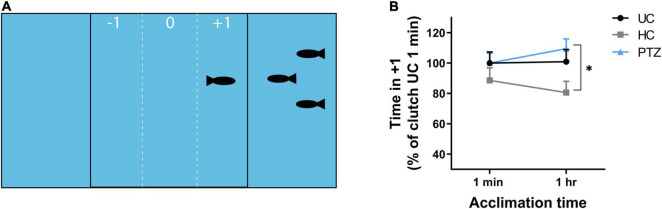
No significant difference in shoaling behavior after seizures, but significantly less shoaling in the handling control (HC) fish. **(A)** The shoaling test apparatus consisted of a tank that was divided by clear panels into three sections. The center section contained the test fish and was marked on the outside to distinguish three preference zones: the side farthest from the target fish (–1), the center (0), and the side closest to the target fish (+1). Positive shoaling was defined as time spent by the test fish in subzone +1. **(B)** The HC test fish shoaled less compared to the pentylenetetrazole (PTZ) test fish after 1-h acclimation to the test apparatus. Data was normalized to the mean of the 1 min untouched controls (UC) group for each clutch to account for clutch to clutch variability. Mixed effects analysis showed a significant main effect of experimental group [*p* = 0.033; *F*_(2,85)_ = 3.554], with *post-hoc* test revealing a significant decrease in the HC group at 1 h compared to the PTZ group (*p* = 0.01). None of the groups differed significantly at the 1-min time point, and the PTZ group was not significantly different from the UC group at either time point. * indicates *p* < 0.05.

In addition to learning deficits, social behavior deficits are another outcome commonly linked to ELS (Lippman-Bell et al., 2013; Dal Canto et al., 2018). To determine whether larval zebrafish seizures delay or alter social behavior development, we assessed shoaling, the likelihood of fish to cluster in social groups (Ledesma and McRobert, 2008; Miller and Gerlai, 2011). If larval seizures caused a developmental delay in social behavior, any deviation from controls would be most stark at the earliest age at which zebrafish begin to show shoaling behavior. Prior studies show a significant increase in shoaling between 26 and 48 dpf (Buske and Gerlai, 2012). Thus, we tested shoaling in 28–32 dpf. After 1 min of acclimation, test fish shoaled for similar amounts of time regardless of experimental group (UC, HC, and PTZ). After 1 h, the PTZ test fish still shoaled as much as the UC fish, but the HC fish shoaled significantly less than the PTZ group. [Mixed-effects analysis showed a significant main effect of experimental group *p* = 0.046; *F*_(2,85)_ = 3.197; *post-hoc* analysis shows a significant decrease of HC at 1 h compared to PTZ at 1 h (*p* = 0.01) with no significant changes between any other groups or time points; Figures 2A,B]. Therefore, we concluded early-life handling, but not ELS, influenced later-life shoaling behavior.

### Handled Controls Displayed Heightened Stress Responses Not Observed in Zebrafish That Experienced Seizures

**FIGURE 3:**
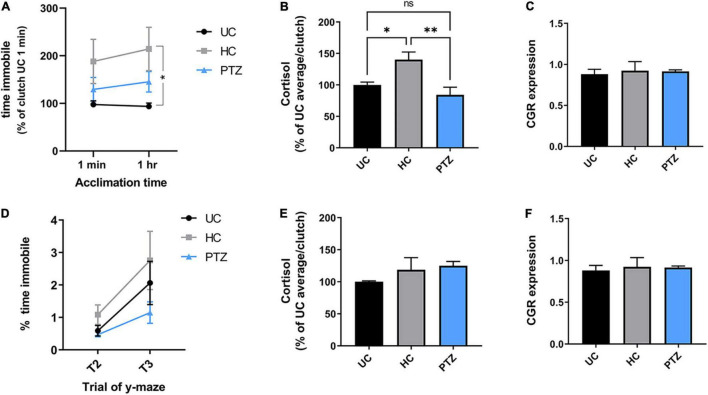
Increased acute stress response in handling control (HC) fish but not pentylenetetrazole (PTZ) fish, at 30 dpf, decreasing by dpf 60. **(A,B)** HC fish showed heightened stress responses at 30 dpf. **(A)** Cumulative time immobile during the shoaling assay [normalized to the untouched controls (UC) 1 min average for each clutch]. Two-way repeated-measures ANOVA showed a significant main effect of treatment group [*p* = 0.037, *F*_(2,139)_ = 3.373]. *Post-hoc* tests showed a significant increase in the HC group at the 1 h time point, as compared to UC (*p* = 0.018), though there was a trending increase in HC compared to UC beginning at 1 min (*p* = 0.098). There was no significant difference between UC and PTZ (*p* = 0.75 at 1 min; *p* = 0.46 at 1 h). **(B)** Cortisol levels in 30 dpf fish immediately after netting were significantly higher in the HC group compared to UC (*p* = 0.02) and PTZ (*p* = 0.004), with no significant difference between PTZ and UC (*p* = 0.30). **(C)** Glucocorticoid receptor mRNA was similar across experimental groups at 30 dpf. **(D,E)** Freezing and cortisol responses returned to levels comparable UC fish by 60 dpf, and **(F)** GCR RNA expression remained similar across groups [Freezing, main effect of experimental groups by 2-way repeated-measures ANOVA *p* = 0.22, *F*_(2,17)_ = 1.655; *n* = 7 UC, 7 HC, 6 PTZ]. * indicates *p* < 0.05; ** indicates *p* < 0.01; ns or unmarked indicates no significance *p* > 0.05.

We next assessed anxiety behaviors by measuring the freezing behavior during the shoaling and y-maze tasks, as freezing in the absence of a predator is thought to be analogous to anxiety (Stewart et al., 2012). All zebrafish showed similar levels of freezing behavior after the first minute of acclimation in the shoaling task. However, once the fish had acclimated to the shoaling tank for 1 h, only zebrafish in the HC group displayed significantly more freezing than the UC group; freezing behavior in the PTZ group did not differ significantly from naïve controls (Figure 3A). [Two-way repeated measures ANOVA showed a main effect of experimental group (*F*_(2,139)_ = 3.373, *p* = 0.037), with a *post-hoc* test showing a significant difference between HC and UC groups at the 1 h acclimation time point; *p* = 0.027].

To confirm that the increased freezing behavior in the HC zebrafish was indicative of a heightened stress response, we next measured cortisol levels in each group after gently netting the fish for 2–3 s. HC fish showed elevated levels of cortisol compared to both the UC and PTZ groups, with no change between the PTZ and UC groups [*p* = 0.008, *F*_(2,12)_ = 7.327 by ANOVA, with *post hoc* analysis showing a significant difference of HC vs. UC (*p* = 0.02) and PTZ (*p* = 0.004); UC vs. PTZ *p* = 0.3; mean = 100.0 ± 6.5 for UC, 144.1 ± 13.53 for HC, and 79.99 ± 14.94 for PTZ; *n* = 5 UC, 6 HC, and 4 PTZ samples with two zebrafish pooled per sample; Figure 3B]. Taken together with the y-maze data, these results indicate that early-life seizures attenuated the development of the heightened stress response after early-life stress seen in handled controls.

Elevated cortisol levels occur with both acute and chronic stress (Manuel et al., 2014). However, chronic stress can be marked by attenuated expression of one of the primary cortisol receptors, the glucocorticoid receptor (Kitraki et al., 1999; Piato et al., 2011). Therefore, to distinguish between an acute stress response and chronically increased stress, we next measured levels of glucocorticoid receptor mRNA. Unlike cortisol levels, glucocorticoid receptor expression remained similar across experimental groups at 30 dpf (Figure 3C). Thus, the elevated cortisol levels in the HC fish likely represent an acute heightened response to the mild stressor and not chronic anxiety.

By 60 dpf, the freezing behavior and the elevated cortisol response in the HC fish returned closer to UC levels, and glucocorticoid receptor mRNA expression remained similar between groups (Figures 3D–F). This return to control levels supports the idea that HC fish did not have chronic anxiety, but a heightened stress response that dissipated over time as the fish lived on the tank system, undisturbed by outside stressors.

### Handled Fish Displayed a Heightened Acoustic Startle Response

A heightened stress response after an early-life stressor, such as handling, can represent a learned, adaptive response (Belda et al., 2008; Berry and López-Martínez, 2020); multiple animal models demonstrate that mild stressors change later behavior to protect the animal from further harm (Finsterwald and Alberini, 2014; Berry and López-Martínez, 2020). If this were the case in the handled larval zebrafish, we would expect the HC group to develop behaviors advantageous to their survival. Therefore, we compared the HC acoustic startle response (ASR) to that of the PTZ and UC groups to measure a potential advantage for escape.

**FIGURE 4:**
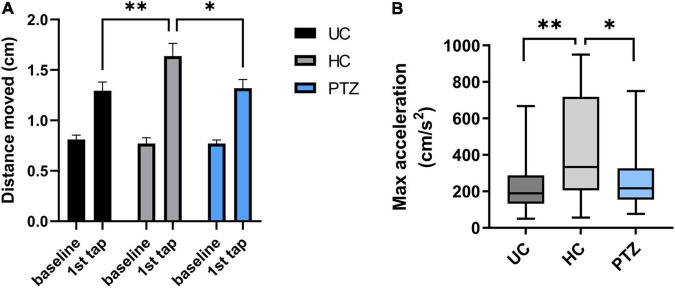
Enhanced acoustic startle response in handling control (HC) fish. The HC group had a more robust reaction to a startling stimulus, indicating that they may have an escape advantage over the other groups. **(A)** Two-way repeated measures ANOVA of distance moved in the first second from the start of the tap stimulus showed a significant effect of tap [*F*_(1,68)_ = 139.5, *p* < 0.0001] and of tap x experimental group [*F*_(2,68)_ = 4.826, *p* = 0.011]. *Post-hoc* Sidak’s multiple comparison test revealed a significant increase between baseline and first tap (*p* < 0.0001) for all three experimental groups, demonstrating that all groups displayed an acoustic startle response (ASR). However, the *post-hoc* test also showed the HC group moved significantly further in response to the tap (in the first second from the start of the tap) compared to both untouched controls (UC) (*p* = 0.007) and pentylenetetrazole (PTZ) (*p* = 0.0141) groups. **(B)**. A Kruskal-Wallis test with a *post-hoc* Dunn’s multiple comparisons test revealed that HC fish also reached a higher max acceleration in the first second from the start of the tap, compared to both UC (*p* = 0.001) and PTZ groups (*p* = 0.029; graph shows median because the data were not normally distributed). * indicates *p* < 0.05; ** indicates *p* < 0.01; ns or unmarked indicates no significance *p* > 0.05.

Zebrafish will show a startle response to a range of stimuli (Colwill and Creton, 2011). To evoke an ASR, zebrafish were acclimated to a 24-well plate in the DanioVision chamber for 25 min, then exposed to a loud tap. The response of each fish was quantified by distance traveled in the first second after the tap. Two-way repeated-measures ANOVA revealed a significant effect of tap x experimental group [*F*_(2,68)_ = 4.826, *p* = 0.011], with *post-hoc* Sidak’s multiple comparison test showing a significant increase between baseline and first tap (*p* < 0.0001) for all three experimental groups. These data indicate that all three experimental groups responded to the acoustic tapping stimulus and displayed an ASR, moving a greater distance than during the pre-tap baseline (Figure 4A). In addition, the 2-way repeated measures ANOVA revealed a significant effect of tapping [*F*_(1,68)_ = 139.5, *p* < 0.0001]. A *post hoc* Sidak’s multiple comparison test showed no differences between groups at baseline [mean distance moved (cm) = 0.81 ± 0.04 for UC, 0.77 ± 0.06 for HC, and 0.77 ± 0.04 for PTZ group], but revealed a significantly increased ASR in the HC group compared to both UC (*p* = 0.007) and PTZ (*p* = 0.014), showing that the HC fish moved significantly farther in the one second from the start of the tap than either of the other two groups [mean distance moved at tap = 1.29 ± 0.09 for UC, 1.64 ± 0.13 for HC, and 1.32 ± 0.09 for PTZ group; *n* = 24 UC, 23 HC, and 24 PTZ]. Notably, the ASR of the PTZ group did not differ from that of the UC group (*p* = 0.995). HC fish also reached a higher max acceleration in the first second from the start of the tap, compared to both UC [*p* = 0.001 by Kruskal-Wallis with a *post hoc* Dunn’s multiple comparisons test] and PTZ groups (*p* = 0.029; Figure 4B). These data demonstrate that the HC group had an enhanced response to a startling stimulus, indicating that they may have an escape advantage over the other groups.

## Discussion

In the current study, we aimed to expand the use of the larval zebrafish PTZ-induced seizure model to examine long-term cognitive and behavioral deficits linked to ELS. We found that after three daily 40-min exposures to PTZ at 5–7 dpf, zebrafish showed less recall in a y-maze learning task later in life compared to naïve controls (UC group), but similar social and anxiety behaviors. Handling control (HC) fish not exposed to PTZ developed an enhanced acute stress response not seen in PTZ fish, as well as a faster escape response than UC and PTZ fish. Thus, we concluded that seizures in larval zebrafish also impaired the development of a heightened stress response that would normally develop after experiencing mild early-life stress to help the fish adapt to future stressors. These findings support the use of a modified larval zebrafish seizure model to study cognitive consequences of ELS, and provide novel information on the long-term outcomes of ELS in zebrafish.

A primary goal of this study was to characterize consequences of larval zebrafish seizures for more general use in the field. Thus, it is important to compare the PTZ-seizure induction model used here to those described in prior studies. Most prior studies are based on Baraban et al.’s (2005) model, exposing 7 dpf zebrafish to 15 mM PTZ. The original description of the model compared multiple concentrations of PTZ. While both 5 and 15 mM PTZ exposure led to equivalent numbers of fish displaying Stage 1 (fast swimming) and Stage 2 (circular swimming) seizures, far fewer fish reached Stage 3 (clonic seizures) (Baraban et al., 2005). Later studies added to these findings, reporting similar seizure intensities reached by 5 dpf fish exposed to 4 to 40 mM PTZ, but with a shorter latency to, and longer cumulative duration of, Stage 2 seizures at higher PTZ concentrations (Bandara et al., 2020). The Stage 2 seizures in the current study are phenotypically identical to Stage 2 seizures described in prior studies, with the characteristic circular swimming, greater distance moved (Baraban et al., 2005), and more high-speed movements (Winter et al., 2017) compared to clutchmates not exposed to PTZ. Based on the comparative studies mentioned above, the seizure induction protocol in the current study, wherein fish were exposed to less PTZ (5 mM), but for 3 consecutive days (5–7 dpf), may induce fewer Stage 3 seizures. However, more Stage 2 seizures occur cumulatively across all 3 days in our model. Although additional studies will be required to address this further, an increased cumulative number of seizures may partially explain why the 3-day seizure model lead to a more profound learning deficit than that seen in our initial studies using the 15 mM PTZ seizure induction paradigm at 7 dpf.

The learning deficits described in the current study after larval seizures are consistent with studies reporting changes in learning and memory after seizures induced in adult zebrafish (Lee et al., 2010; Canzian et al., 2019). Zebrafish capacity for learning and memory is broad and well-studied. Multiple investigators have demonstrated zebrafish competence in tasks assessing spatial learning, habituation, and fear-conditioned learning (Best et al., 2008; Randlett et al., 2019; Yashina et al., 2019). Here, we found a deficit in y-maze performance, which assesses spatial memory, in adult fish after larval seizures. It is still unclear whether seizures in larval zebrafish only affect spatial memory or whether other forms of memory are also impaired, though the lack of development of the heightened stress response implies that other forms of memory might also be impaired. Additional studies will be required to address this issue and test spatial learning in other apparatuses. A caveat of the y-maze used without fear conditioning paradigms is that only adult zebrafish could recall the novel arm consistently. Thus the current study did not assess memory in younger zebrafish. Therefore, the current study leaves open the question of when the memory deficits begin. Future studies will examine the timing of learning deficit onset through tasks that can more consistently measure learning in larval zebrafish.

The post-ELS learning deficits described here may provide a useful framework in which to investigate molecular mechanisms that link ELS to cognitive consequences, to eventually achieve the goal of predicting and preventing these deficits. Multiple molecular pathways and neural circuits have been linked to cognitive consequences of ELS in rodent models. Many of these involve hippocampal abnormalities as well as changes in NMDA and AMPA glutamate receptors (Brooks-Kayal, 2011; Nishimura et al., 2011; Talos et al., 2012; Lippman-Bell et al., 2013; Casanova et al., 2014; Bernard and Benke, 2015). Similar to rodents, memory acquisition in the zebrafish y-maze is at least in part dependent on NMDA receptor function, as it can be blocked with the NMDA receptor antagonist MK-801 (Cognato Gde et al., 2012). Interestingly, another form of learning and memory, classical conditioning, has recently been shown to dynamically regulate excitatory synapse number in two specific areas of the zebrafish brain, the lateral and medial pallium (Dempsey et al., 2022). These telencephalic regions are proposed to be analogous to the mammalian hippocampus and amygdala, respectively (Salas et al., 2006; Ganz et al., 2014; Calvo and Schluessel, 2021; Gerlach and Wullimann, 2021), and thus the lateral pallium is likely contain neural circuits for spatial learning as well. Supporting this, goldfish with lateral pallium lesions show deficits in spatial learning (Rodríguez et al., 2002). Taken together with rodent studies demonstrating the role of postsynaptic glutamate receptors in the consequences of ELS (Lippman-Bell et al., 2013), these data indicate that glutamatergic synapses within the zebrafish pallium would be a worthwhile focus for future studies investigating the mechanisms linking larval seizures to memory deficits in zebrafish.

In addition to their capacity for learning and memory, zebrafish also demonstrate well-characterized social behaviors. Like most fish species studied, zebrafish spend their time in simple social aggregations known as shoals (Miller and Gerlai, 2011). The benefits of shoaling include increased foraging success, access to potential mates, and possible protection from predators (Pitcher, 1986; Krause et al., 2002). As a behavior, shoaling is well-documented in the literature, and usually involves placing fish into a situation in which they demonstrate shoaling preferences. Typically, fish chose to swim near other groups of fish, rather than near empty chambers. Further, when given choices between possible shoal-mates, they choose fish that are phenotypically similar to themselves, possibly gaining advantages from a phenomenon known as the Confusion Effect, in which predators have difficulty targeting individual prey within homogeneous groups (Neill and Cullen, 1974; Ohguchi, 1981; Landeau and Terborgh, 1986). Studies on *D. rerio* show they choose to join shoals, and will discriminate between potential shoal-mates based on body pattern (Rosenthal and Ryan, 2005), sex (Ruhl and McRobert, 2005), and shoal size (Pritchard et al., 2001). Based on the complexity of zebrafish shoaling choice, it is important to note that the shoaling task performed in the current study measures social preference, but not, for example, preference for social novelty, an aspect of social behavior impaired in rats following ELS (Lippman-Bell et al., 2013). Thus, although the current study demonstrates that seizures do not impair overall sociability of the zebrafish, there may be more subtle aspects of zebrafish social behavior altered by ELS not examined here.

Compared to studies of cognitive and behavioral deficits following seizures in adult zebrafish, the current study may indicate that seizures in larval zebrafish may cause fewer impairments, even with the modified multi-day seizure induction model. In adult zebrafish, PTZ-induced seizures lead to increased anxiety behaviors and decreased sociability not observed in the current study (Canzian et al., 2019). However, the studies performed after seizures induced in adult zebrafish assessed the behavioral changes shortly (within 24 h) after the seizures occurred. Thus, it is possible that larval zebrafish also show these changes acutely but recover over time, in which case the more chronic post-seizure time points assessed in the current study would not have captured the changes. Another possible difference between the current study and published studies of adult PTZ-induced seizures may be due to the interaction between anxiety and the mechanism of action of PTZ. Because PTZ acts as a non-competitive antagonist of the GABA_*A*_ receptor (Hansen et al., 2004), and GABA_*A*_ is involved in anxiety (Möhler, 2012), it is possible that the anxiety behaviors seen shortly after PTZ are due more to the PTZ itself than to the seizures it provokes. If this is the case, the lack of a post-seizure change in anxiety behaviors observed in the current study could result from assessing behavioral outcomes at time points well after PTZ exposure, after direct effects of PTZ on the GABA_*A*_ receptor would have resolved.

The idea that PTZ itself may affect post-seizure outcomes highlights the importance of identifying confounding factors in the quickly expanding zebrafish seizure model. For example, in the current study, we found that basic handling in larval zebrafish affects behavioral development. Because the current study employed a modified PTZ-induction model that occurred over 3 days instead of one, the fish experienced more handling than would occur with the 1-day PTZ model. However, at this age of rapid development, the general effects of handling may need to be considered when analyzing future studies of larval zebrafish behavior.

## Conclusion

The current study provides novel evidence that seizures induced in larval zebrafish can lead to long-term cognitive changes, impairing learning and the development of an adaptive heightened stress response after mild early-life stress. These findings provide an important addition to the existing larval zebrafish seizure models, supporting the use of larval zebrafish for future research into the cognitive consequences of ELS.

## Data Availability Statement

The raw data supporting the conclusions of this article will be made available by the authors, without undue reservation.

## Ethics Statement

The animal study was reviewed and approved by Philadelphia College of Osteopathic Medicine Institutional Animal Care and Use Committee (IACUC), and Saint Joseph’s University IACUC.

## Author Contributions

HS: investigation, conceptualization, methodology, analysis, and writing. AR: investigation, methodology, analysis, and writing. DF and KB: investigation. SM: methodology, supervision, writing, resources, and funding acquisition. JL-B: conceptualization, methodology, supervision, writing, resources, visualization, funding acquisition, and project administration. All authors contributed to the article and approved the submitted version.

## Conflict of Interest

The authors declare that the research was conducted in the absence of any commercial or financial relationships that could be construed as a potential conflict of interest.

## Publisher’s Note

All claims expressed in this article are solely those of the authors and do not necessarily represent those of their affiliated organizations, or those of the publisher, the editors and the reviewers. Any product that may be evaluated in this article, or claim that may be made by its manufacturer, is not guaranteed or endorsed by the publisher.
