# Supplementary material for: Learning Deficits and Attenuated Adaptive Stress Response After Early-Life Seizures in Zebrafish
Source: Front Neurosci. 2022 Apr 22;16:869671. doi: 10.3389/fnins.2022.869671 (PMC9073075; doi:10.3389/fnins.2022.869671)
Supplement: Supplementary file 1 [file Data_Sheet_1.docx]

**Supplemental Materials for Singh et al.**


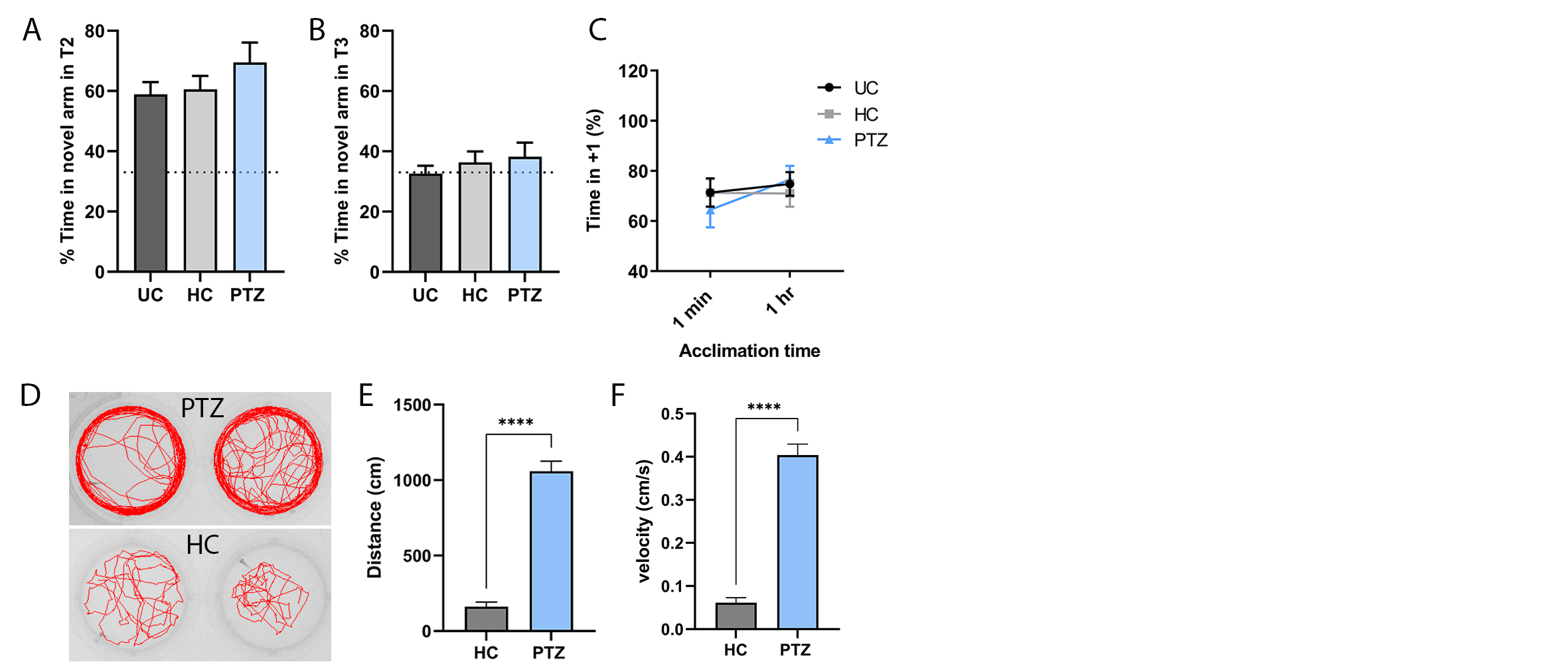


**Supplemental Figure 1: Rationale for 3-day seizure model.** We performed pilot studies using the previously described 15mM PTZ seizure model in 7 dpf zebrafish, but did not find a significant change in performance in either the y-maze (A,B) or in the shoaling task (C) compared to the control groups. A) All zebrafish tested at 2.5-3 months of age, regardless of experimental group, showed a preference for the novel arm in T2 of the y-maze task. Dotted line marks 33%. B) There was no change across groups during recall phase T3 after a 60-minute inter-trial interval. (n=8 UC, 17 HC, and 12 PTZ; mean ± SE = 32.6 ± 2.6% for UC, 36.4 ± 3.6% for HC, and 38.3 ± 4.7% for PTZ; one-way ANOVA p=0.69, F (2, 34) = 0.38). C) There was no change in shoaling across experimental groups in 4-week old zebrafish. (Shoaling at 1 minute (some videos could not be analyzed due to recording errors), n= 20 UC, 26 HC, 21 PTZ; mean ± SE = 71.38 ± 5.7% for UC; 71.28 ± 5.4% for HC, 64.47 ± 7.0% for PTZ; Shoaling at 1 hour, n= 24 UC, 29 HC, 23 PTZ; mean ± SE = 74.8 ± 4.7% for UC, 71.02 ± 5.3% for HC, 76.54 ± 5.5% for PTZ.) Mixed effects analysis confirmed no effect of experimental group (p=0.95, F (2, 73) = 0.05).


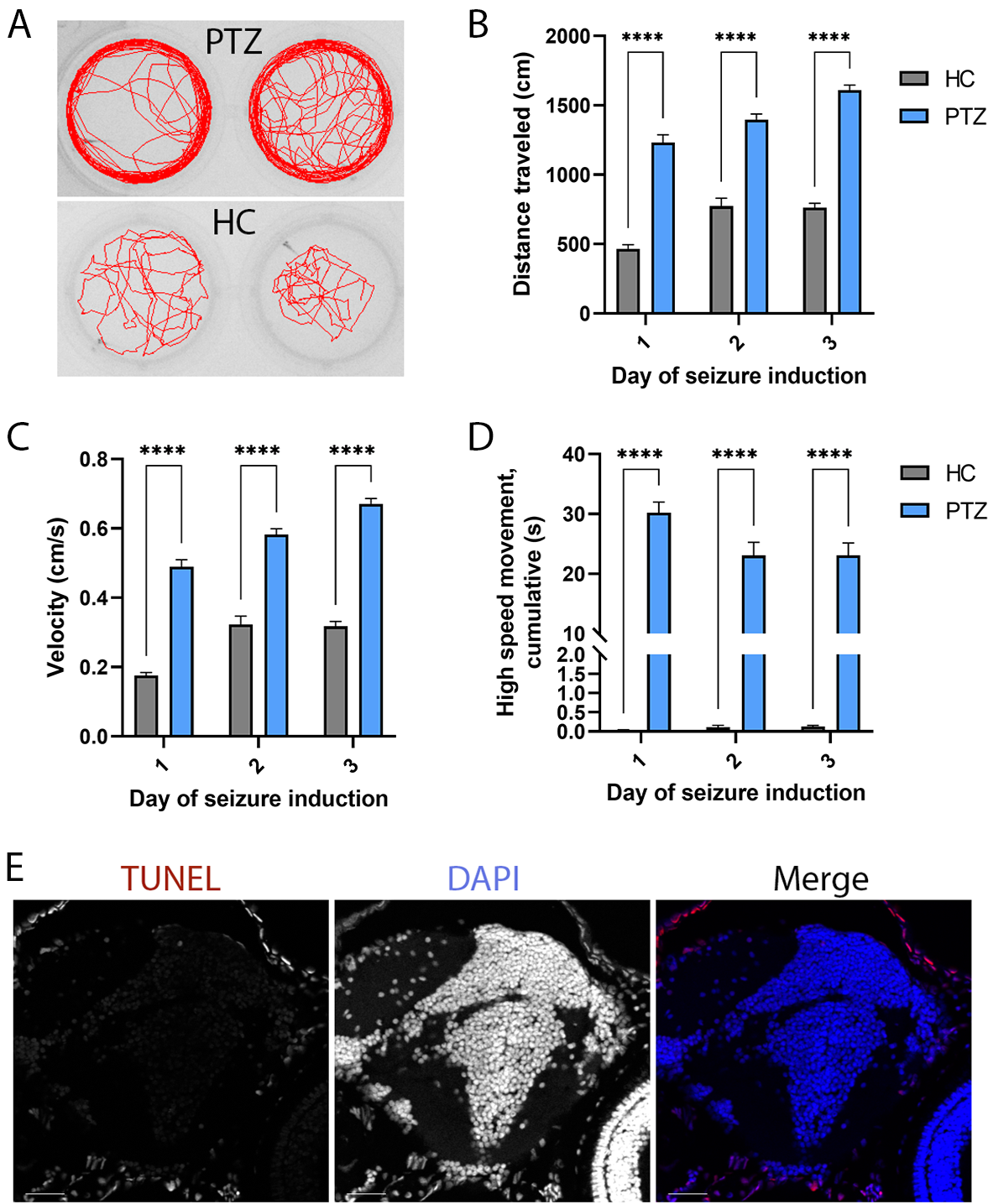


**Supplemental Figure 2: Characterization of 3-day seizure model.** Fish exposed to 5mM PTZ for 40 minutes per day from 5-7 dpf show Stage 2 seizures similar to previously in single day larval zebrafish seizure models, shown by A) presence of circular patterns of swimming not seen in handled controls (HC) not exposed to PTZ, B) increased total distance travelled, C) velocity, and D) percent of time spent performing high speed movements, as compared to HC fish. High speed movements were defined as movements ≥ 2cm/s, as described by other groups quantifying larval seizure behavior (Winter et al., 2017). The latter parameter is important as hyper-locomotion alone can fall under the classification of Stage 1 seizures, whereas Stage 2 includes “rapid movement” in addition to the characteristic circular swimming (Bandara et al., 2020). Statistical significance was tested for the parameters shown in B-D by two-way ANOVA. There was a significant effect of experimental group for all parameters, with post-hoc tests confirming the statistically significant differences between HC and PTZ fish on each day of the experiment (for B, p<0.0001, F (1, 138) = 446.3, post-hoc p<0.0001; for C, p<0.0001, F (1, 137) = 488.3), post-hoc p<0.0001; for D, p<0.0001, F (1, 138) = 483.0, post-hoc p<0.0001). E) The 3-day seizure induction protocol does not cause extensive cell death in the brain, as seen by TUNEL staining in 8 dpf zebrafish, 24 hours after the final day of seizure induction. The dorsal region pictured here contains the pallidum, as determined by comparisons to the Bio-Atlas (http://bio-atlas.com/zebrafish/) plate 3 of the 4 dpf transverse (rostral to caudal) labeled sections, and published descriptions (Messina et al., 2022). The pallium is a telencephalic region that contains areas (lateral and medial pallium) thought to be homologous to the mammalian hippocampus and amygdala (Ganz et al., 2014; Calvo and Schluessel, 2021; Gerlach and Wullimann, 2021).


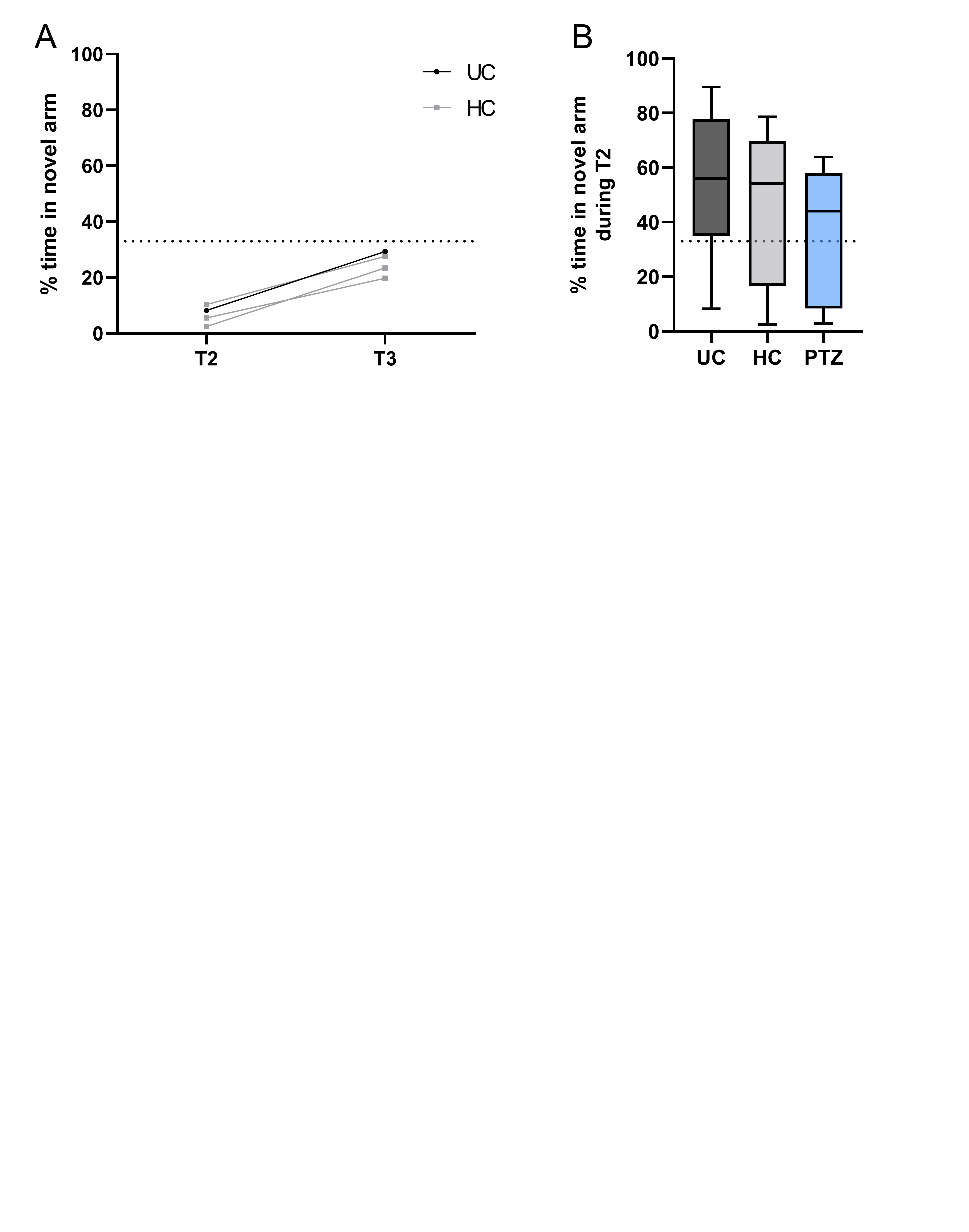


**Combined**

**Supplemental Figure 3: Rationale for y-maze inclusion criteria**

A) Recall in the y-maze task is measured by time spent in the novel arm in Trial 3. No control fish (either UC or HC, n=4) that spent less than 15% of T2 in the novel arm spent the majority (>33%, marked by the dotted line) of T3 in the novel arm. B) The PTZ group demonstrated a slight, though non-significant, decrease in time spent in the novel arm in T2 (p=0.28 by Kruskal-Wallis test). Because a decreased novelty preference could confound the recall data, only fish that spent at least 15% of T2 in the novel arm were included in the study. This excluded 1 UC, 3 HC, and 4 PTZ fish.

| nr3c1 – Forward | 5’ ACAGCTTCTTCCAGCCTCAG 3’ |
| --- | --- |
| nr3c1 – Reverse | 5’ CCGGTGTTCTCCTGTTTGAT 3’ |
| elf1a – Forward | 5’ CTTCTCAGGCTGACTGTGC 3’ |
| elf1a – Reverse | 5’ CCGCTAGCATTACCCTCC 3’ |

**Supplemental Table 1: Primer sequences.** Reverse and forward primers for the gene of interest Nuclear Receptor Subfamily 3, Group C, Member 1 (glucocorticoid receptor; nr3c1), and housekeeping gene, elf1a.

References for Supplemental Materials:

Bandara, S.B., Carty, D.R., Singh, V., Harvey, D.J., Vasylieva, N., Pressly, B., et al. (2020). Susceptibility of larval zebrafish to the seizurogenic activity of GABA type A receptor antagonists. *Neurotoxicology* 76**,** 220-234. doi: 10.1016/j.neuro.2019.12.001.

Calvo, R., and Schluessel, V. (2021). Neural substrates involved in the cognitive information processing in teleost fish. *Anim Cogn* 24(5)**,** 923-946. doi: 10.1007/s10071-021-01514-3.

Ganz, J., Kroehne, V., Freudenreich, D., Machate, A., Geffarth, M., Braasch, I., et al. (2014). Subdivisions of the adult zebrafish pallium based on molecular marker analysis. *F1000Res* 3**,** 308. doi: 10.12688/f1000research.5595.2.

Gerlach, G., and Wullimann, M.F. (2021). Neural pathways of olfactory kin imprinting and kin recognition in zebrafish. *Cell Tissue Res* 383(1)**,** 273-287. doi: 10.1007/s00441-020-03378-4.

Messina, A., Potrich, D., Schiona, I., Sovrano, V.A., Fraser, S.E., Brennan, C.H., et al. (2022). Neurons in the dorso-central division of zebrafish pallium respond to change in visual numerosity. *Cerebral Cortex* 32(2)**,** 418-428.

Winter, M.J., Windell, D., Metz, J., Matthews, P., Pinion, J., Brown, J.T., et al. (2017). 4-dimensional functional profiling in the convulsant-treated larval zebrafish brain. *Sci Rep* 7(1)**,** 6581. doi: 10.1038/s41598-017-06646-6.
